# PD171 National Health Service Wales - First UK Nation To Approve Dostarlimab For The Treatment Of Rectal Cancer

**DOI:** 10.1017/S0266462324003970

**Published:** 2025-01-07

**Authors:** Rachel Jonas, Rosie Spears, Gail Woodland, James Coulson

## Abstract

**Introduction:**

Dostarlimab is a monoclonal antibody that blocks the programmed cell death protein 1 receptor. Emerging evidence has shown complete clinical response with dostarlimab in patients with locally advanced, treatment-naïve stage II or III rectal cancer where the tumor is either mismatch repair deficient or has high microsatellite instability. Dostarlimab has the potential to replace standard treatments such as chemoradiotherapy and surgery.

**Methods:**

As part of the One Wales medicines assessment process, a literature search was performed and the marketing authorization holder was contacted to ensure the most up-to-date information was available. Clinical experts were consulted to advise where the medicine would sit within current therapy pathways in National Health Service Wales and to ensure that outcome data could be collected if dostarlimab were approved. After reviewing the evidence, an evidence summary report was written by the All Wales Therapeutics and Toxicology Centre that included the clinical and cost effectiveness, safety, and budget impact of dostarlimab.

**Results:**

The One Wales Medicines Advisory Group assessed the evidence in June 2023, recommending access to dostarlimab for patients meeting the criteria for treatment. This recommendation was endorsed by the All Wales Medicines Strategy Group and ratified by the Welsh Government in August 2023. Starting and stopping criteria for dostarlimab were developed in collaboration with clinical experts to complement the One Wales decision. All patients in Wales who meet the agreed starting criteria will now be given the option for routine treatment with dostarlimab. To date, two patients have started treatment with dostarlimab, both of whom have reported treatment response at three months.

**Conclusions:**

Wales is the first nation in the UK to approve routine access to dostarlimab for rectal cancer. The One Wales process allows access to dostarlimab, with the associated potential for avoiding life-changing surgery. Monitoring patient outcomes will provide real world data to enhance the immature dataset currently available on the clinical and cost effectiveness of dostarlimab for rectal cancer.

